# Patient Safety Culture: The Impact on Workplace Violence and Health Worker Burnout

**DOI:** 10.1177/21650799221126364

**Published:** 2022-12-08

**Authors:** Sinhye Kim, Rebecca Kitzmiller, Marianne Baernholdt, Mary R. Lynn, Cheryl B. Jones

**Affiliations:** 1 The University of North Carolina at Chapel Hill; 2 The University of Virginia School of Nursing

**Keywords:** patient safety culture, workplace violence, burnout, worker safety, health care workers

## Abstract

**Background:**

Patient and health care worker safety is an interconnected phenomenon. To date, few studies have examined the relationship between patient and worker safety, specifically with respect to work safety culture. Therefore, we examined patient safety culture, workplace violence (WPV), and burnout in health care workers to identify whether patient safety culture factors influence worker burnout and WPV.

**Methods:**

This cross-sectional study used secondary survey data sent to approximately 7,100 health care workers at a large academic medical center in the United States. Instruments included the Hospital Survey on Patient Safety Culture, a WPV scale measuring physical and verbal violence perpetrated by patients or visitors, and the Emotional Exhaustion scale from the Maslach Burnout Inventory.

**Findings:**

These analyses included 3,312 (47%) hospital staff who directly interacted with patients. Over half of nurse (62%), physician (53%), and allied health professional respondents (52%) reported experiencing verbal violence from a patient, and 39% of nurses and 14% of physicians reported experiencing physical violence from a patient. Burnout levels for nurses (2.67 ± 1.02) and physicians (2.65 ± 0.93) were higher than the overall average for all staff (2.61 ± 1.0). Higher levels of worker-reported patient safety culture were associated with lower odds of WPV (0.47) and lower burnout scores among workers (*B* = −1.02). Teamwork across units, handoffs, and transitions were dimensions of patient safety culture that also influenced WPV and burnout.

**Conclusions/Application to Practice:**

Our findings suggest that improvements in hospital strategies aimed at patient safety culture, including team cohesion with handoffs and transitions, could positively influence a reduction in WPV and burnout among health care workers.

## Background

The safety of patients and health care workers in a health care system is not independent; in fact, they may be mutually affected (The Joint Commission, 2012). As most health care services are provided by health care workers, maintaining the health and safety of these individuals is critical to ensuring the quality of care. However, many health care workers experience physical and psychological harm when providing patient services (Loeppke et al., 2017). The recent pandemic has elevated concerns regarding the relationship between patient and worker safety and provides an opportunity to reassess health care institutions’ capacity to ensure and enhance the safety and health of workers (McGaffigan et al., 2020; Shaw et al., 2020). Various leading health organizations have published guidelines to respond to these challenges and opportunities.

The World Health Organization (2020) announced that the theme of World Patient Safety Day 2020 was “Health worker safety: a priority for patient safety,” calling for the integration of policies and strategies that promote worker health and patient safety, the development and implementation of national programs for the health and safety of workers, the protection of workers from workplace violence (WPV), the prioritization of mental health and psychological well-being of workers, and the protection of workers from physical and psychological risks. The Institute for Healthcare Improvement (IHI) also highlighted a total-systems approach to safety in which all stakeholders cooperate to ensure that safe care is delivered to patients and that harm to patients and those who care for them is minimized (National Steering Committee for Patient Safety [NSC], 2020). Both guidelines emphasize the need for an integrative approach to patient and worker safety and the establishment of a safety culture that promotes safety awareness and behaviors.

A culture of safety in health care refers to an organization’s fundamental values, norms, and expectations regarding the safety and protection of both patients and workers from harm and injuries (U.S. Department of Labor [U.S. DOL], n.d.). Workers’ attitudes and behaviors contributing to their organization’s ongoing safety performance are influenced by their employer’s safety culture (Morello et al., 2013). A safer environment for both patients and health workers is embedded in the organizational culture and connected to the institutional and structural systems (U.S. DOL, n.d.). Exploring common cultural characteristics that affect the health and safety of patients and workers may provide a foundation for developing an integrated workplace safety strategy.

Patient safety culture assessment is encouraged by national health policymakers, with hospitals in many countries regularly conducting patient safety culture surveys (Agency for Healthcare Research and Quality [AHRQ], 2019). These surveys provide opportunities for health organizations to evaluate and systematically improve their patient safety culture. Identifying patient safety culture attributes that affect health workers can support the development of an effective integrated strategy that benefits both patients and workers by improving an organization’s overall safety culture.

### Patient Safety Culture and WPV

Workplace violence is a serious concern that threatens the health care work environment, including the safety and health of health care workers in hospitals (Phillips, 2016). Patients are the most common perpetrators of violence toward hospital staff and are the primary cause of violence-related injuries requiring employee absences from work (U.S. Bureau of Labor Statistics, 2020). Research has identified multiple factors that contribute to violence toward health care workers, including perpetrator characteristics, job conditions, and work environment (Edward et al., 2014; Pompeii et al., 2020). Although WPV is due, in part, to a structural issue influenced by the system and culture of the organization (International Labour Office/International Council of Nurses/World Health Organization/Public Services International, 2002), research on these relationships has only recently been acknowledged.

According to previous studies, a strong safety culture is associated with a positive patient experience (Abrahamson et al., 2016; Smith et al., 2017) and patient perceptions of satisfaction (Mazurenko et al., 2019). Patients’ negative hospital experiences and low patient satisfaction scores are predictors of aggressive/violent behavior toward health care workers (Gudde et al., 2015; Hahn et al., 2010). The organizational safety culture may therefore be a key factor in WPV prevention. However, to our knowledge, no studies have explored the relationship between patient safety culture and WPV. Identifying specific aspects of patient safety culture associated with WPV incidents could aid the development of preventive strategies and improvement of organizational culture to mitigate WPV risk.

### Patient Safety Culture and Burnout

Job burnout is an individual’s emotional response to work stress (Maslach et al., 2001). An insufficient workforce, excessive workload, time pressure, and unstable and unpredictable patient health conditions place emotional demands on health care workers and contribute to burnout (Maslach & Leiter, 2016). Previous studies have shown that burnout is significantly related to health care workers’ well-being, job satisfaction, and job retention and affects their job engagement and performance (Dall’Ora et al., 2020; Janes et al., 2021). Burnout also threatens workers’ perceptions of their safety and is linked to reduced safety behaviors, including underreporting of adverse safety events, which in turn affect patient safety (Halbesleben et al., 2008; Vogus et al., 2020). Adequate burnout management is crucial for maintaining patients’ and workers’ safety and health and the organization’s efficient operation.

Efforts to improve patient safety culture may also affect the safety and health of health care workers. Organizations with strong patient safety culture are characterized by open communication based on reciprocal trust, common perceptions about the value of safety, and confidence in the effectiveness of prevention initiatives (Sorra et al., 2016). Such organizations seek to provide employees with transparent and clear work procedures to enhance patient safety and improve work systems to minimize work-related errors and failures (Brigham et al., 2018; Kath et al., 2010). These activities may contribute to improved patient safety and reduced workload and stress on health care workers by enhancing their working environment.

The overall goal of this study was to examine the relationships among patient safety culture, WPV, and burnout in health care workers. Specifically, we aimed to (a) examine the levels of patient safety culture, WPV, and burnout by occupation; (b) identify patient safety culture factors that contribute to WPV perpetrated by patients or visitors; and (c) identify patient safety culture factors that contribute to health care workers’ burnout. The findings from this study can inform organizational leaders and occupational health professionals on the development of practical solutions to improve health care workers’ safety and health by promoting patient safety culture.

## Methods

This cross-sectional, descriptive study used secondary data gathered in 2017 from hospital staff at a large academic medical center in the southeastern United States. All hospital staff (approximately *N* = 7,100) were invited to participate in an online survey gathering data on patient safety culture, WPV, burnout, and job characteristics (types of occupation, types of work unit, duration working on current unit and in current occupation, and number of hours worked per week). A total of 3,601 staff from 120 units across the hospital responded, for an overall response rate of approximately 51%. Only hospital staff who had contact or direct interactions with patients were included in the current study (*N* = 3,132). Respondents were categorized into four groups: nurses, physicians, allied health professionals, and others. Allied health professionals were classified based on the definition provided by the Association of Schools of Allied Health Professions (2015) and included pharmacists, licensed practical nurses, patient care assistants, respiratory therapists, physical/occupational/speech therapists, technicians/technologists, and administration/management. As this study used existing data, including de-identified information, it was exempt from human subjects review per the University of North Carolina at Chapel Hill’s Institutional Review Board (No. 20-3703).

### Patient Safety Culture

Patient safety culture was measured using 42 items from the AHRQ Hospital Survey on Patient Safety Culture (HSOPS; Sorra et al., 2016), which is composed of 12 dimensions of safety culture (communication openness, handoffs and transitions, nonpunitive response to errors feedback, etc.). The responses are graded on a 5-point Likert-type scale (1 = *strongly disagree* to 5 = *strongly agree*). The score was calculated by averaging each domain’s items, ranging from 1 to 5, with higher average scores indicating a better patient safety culture. Cronbach’s alpha for the total scale was .96, with the individual dimensions ranging from .71 to .88 in the current study.

### WPV

A WPV scale was developed by the medical center to measure three forms of WPV: physical violence from a patient, verbal violence from a patient, and verbal violence from a visitor (Kim et al., 2021). Participants were asked whether they had experienced all three forms of WPV in the past 3 months. Items included the following: “Regardless of the patient’s mental status or medical history, in the past 3 months, how many times have you experienced physical violence (e.g., been hit, grabbed, bitten, scratched) from a patient?” and “Regardless of the patient’s mental status or medical history, in the past 3 months, how many times have you experienced verbal violence (e.g., insults, threats, screaming, cursing) from a patient/a visitor?” Each item was rated on a 4-point scale as follows: 1 (*none*), 2 (*1–5 times*), 3 (*6–10 times*), and 4 (*more than 11 times*). For this study, each type of WPV was formatted as a binary variable (not experienced/experienced).

### Burnout

The four-item Emotional Exhaustion scale from the Maslach Burnout Inventory (Maslach et al., 2001) was used to measure burnout. The validity and reliability of the scale were proved in previous research (Block et al., 2013; Sexton et al., 2014). The survey’s four items were as follows: (a) “I feel fatigued when I get up in the morning and have to face another day on the job,” (b) “I feel burned out from my work,” (c) “I feel frustrated by my job,” and (d) “I feel I am working too hard at my job.” Items were rated on a 5-point Likert-type scale ranging from 1 (*strongly disagree*) to 5 (*strongly agree*), with a higher score indicating a greater level of burnout. The total mean score of the four items was used for analysis. Cronbach’s alpha was .89 in this study, compared with .85 in the study by Sexton et al. (2014).

### Data Analysis

Descriptive analyses were conducted to characterize the health care workers in the study sample and the primary study variables. A one-way analysis of variance with Scheffe post hoc test was conducted to determine whether patient safety culture and burnout levels differed by occupation type. A chi-square test was carried out to investigate whether WPV experiences differed by occupation. Regression analyses were conducted to determine patient safety culture factors that affected health care workers’ safety and health, adjusting for respondents’ job characteristics. Logistic regression analysis was used to predict the likelihood of health care workers experiencing WPV specific to different aspects of patient safety culture, and odds ratios (ORs) and 95% confidence intervals (CIs) were calculated. Multiple linear regression analysis was performed to assess the impact of patient safety culture factors on health care workers’ burnout, and coefficients, *t*-statistics and *p* values were calculated. As WPV was found to be correlated with burnout in our prior study (Kim et al., 2021), we included WPV as a covariate in the multiple regression. Statistical analyses were conducted using IBM SPSS Statistics (Version 27).

## Results

### Sample Characteristics

The sample of this study included 3,132 hospital staff who had contact or direct interactions with patients (Table 1). More than half of the respondents were nurses (56.42%) and the largest number of respondents worked on a general medical unit (16.35%) or the intensive care unit (13.76%). For all occupations, the number of years of work experience in their current work unit and the number of years in their current profession/specialty commonly ranged from 1 to 5 years (48.42% and 34.0%, respectively). Most nurses reported working an average of 20 to 39 hours per week (67.63%), while approximately half of the physicians worked between 40 and 59 hours per week (44.85%).

**Table 1.** Occupational Characteristics of Health Care Worker Participants (*n* = 3,132)

Characteristics	Total (*N* = 3,132)		Nurses (*n* = 1,767)		Physicians (*n* = 273)		Allied health professionals (*n* = 759)		Others (*n* = 333)	
	*n*	%	*n*	%	*n*	%	*n*	%	*n*	%
Unit type										
Medicine	512	16.35	338	19.13	24	8.79	106	13.97	44	13.21
Surgery	366	11.69	225	12.73	10	3.66	91	11.99	40	12.01
Perioperative surgery	296	9.45	164	9.28	68	24.91	41	5.40	23	6.91
Obstetrics	189	6.03	136	7.70	29	10.62	13	1.71	11	3.30
Pediatrics	177	5.65	125	7.07	16	5.86	22	2.90	14	4.20
Emergency department	214	6.83	119	6.73	27	9.89	44	5.80	24	7.21
Intensive care	431	13.76	377	21.34	8	2.93	27	3.56	19	5.71
Psychiatry	205	6.55	110	6.23	29	10.62	40	5.27	26	7.81
Other	742	23.69	173	9.79	62	22.71	375	49.41	132	39.64
Years of working experience in the current unit										
<1	572	18.49	301	17.21	33	12.18	179	23.83	59	18.27
1–5	1,498	48.42	875	50.03	99	36.53	373	49.67	151	46.75
6–10	535	17.29	314	17.95	57	21.03	99	13.18	65	20.12
11–15	245	7.92	132	7.55	33	12.18	61	8.12	19	5.88
16–20	142	4.59	75	4.29	27	9.96	23	3.06	17	5.26
21+	102	3.30	52	2.97	22	8.12	16	2.13	12	3.72
Years of working experience in the current profession/specialty										
<1	244	7.86	142	8.09	17	6.32	61	8.08	24	7.41
1–5	1,055	34.00	647	36.87	75	27.88	251	33.25	82	25.31
6–10	656	21.14	391	22.28	53	19.70	146	19.34	66	20.37
11–15	391	12.60	189	10.77	35	13.01	117	15.50	50	15.43
16–20	309	9.96	157	8.95	29	10.78	71	9.40	52	16.05
21+	448	14.44	229	13.05	60	22.30	109	14.44	50	15.43
Number of hours worked per week										
<20	186	5.97	100	5.67	30	11.03	49	6.52	7	2.14
20–39	1,667	53.52	1,193	67.63	46	16.91	318	42.29	110	33.64
40–59	1,133	36.37	447	25.34	122	44.85	363	48.27	201	61.47
60–79	107	3.43	17	0.96	66	24.26	20	2.66	4	1.22
80+	22	0.70	7	0.40	8	2.94	2	0.27	5	1.53

*Note.* Allied health professionals included pharmacists (*n* = 58), licensed practical nurses (*n* = 5), patient care assistants (*n* = 354), respiratory therapists (*n* = 78), physical/occupational/speech therapists (*n* = 9), technicians/technologists (*n* = 191), and administration/managements (*n* = 64). Others included those who work as unit assistants/clerks/secretaries or in environmental services, patient transportation, service response centers, and guest services.

Table 2 shows the perceptions of patient safety culture, levels of burnout, and WPV experiences by occupation. Analysis of variance revealed that perception of patient safety culture was significantly different by occupation, *F*(3, 3125) = 3.87; *p* = .009. Physicians’ perceptions of patient safety culture (*M* = 3.61, *SD* = 0.59) were lower than those of nurses (*M* = 3.73, *SD* = 0.62) and allied health professionals (*M* = 3.74, *SD* = 0.56).

**Table 2.** Patient Safety Culture, Workplace Violence, and Burnout by Job Title (*n* = 3,132)

Variable	Total	Nurses	Physicians	Allied health professionals	Others
		*M* ± *SD* or *n* (%)			
Patient safety culture^a^	3.72 ± 0.60	3.73_a_ ± 0.62	3.61_a,b_ ± 0.59	3.74_b_ ± 0.56	3.69 ± 0.61
Workplace violence					
Physical violence from patient					
Experienced	987 (32.16)	673 (38.68)	37 (13.70)	205 (27.85)	72 (22.29)
Not experienced	2,082 (67.84)	1,067 (61.32)	233 (86.30)	531 (72.15)	251 (77.71)
Verbal violence from patient					
Experienced	1,749 (56.47)	1,073 (61.17)	143 (52.96)	392 (52.41)	141 (43.38)
Not experienced	1,348 (43.53)	681 (38.83)	127 (47.04)	356 (47.59)	184 (56.62)
Verbal violence from visitor					
Experienced	1,113 (35.91)	737 (41.95)	83 (30.86)	195 (26.00)	98 (30.34)
Not experienced	1,986 (64.09)	1,020 (58.05)	186 (69.14)	555 (74.00)	225 (69.66)
Burnout^a^	2.61 ± 1.00	2.67_a_ ± 1.02	2.65 ± 0.93	2.52_a_ ± 0.98	2.50 ± 1.00

*Note.* Means in a row sharing subscripts are significantly different from each other.

a Range: 1 point (*strongly disagree*) to 5 points (*strongly agree*).

Of the three forms of WPV, verbal violence from a patient was reported most frequently (56.47%), followed by verbal violence from a visitor (35.91%) and physical violence from a patient (32.16%). A chi-square test of WPV by occupation determined that nurses most frequently experienced all three forms of violence. Physical violence experiences were highest among nurses (38.68%) and lowest among physicians (13.7%). More than half of nurses (61.17%), physicians (52.96%), and allied health professionals (52.41%) had experienced verbal violence from a patient. Experiences of verbal violence from a visitor were highest among nurses (41.95%) and lowest among allied health professionals (26.0%).

Burnout level was also significantly different according to occupation, *F*(3, 3119) = 5.45, *p* = .001. Specifically, physician and nurse burnout levels were higher than the overall average. Post hoc analyses using Scheffe’s method also indicated that nurses’ burnout level (*M* = 2.67, *SD* = 1.02) was significantly higher than that of allied health professionals (*M* = 2.52, *SD* = 0.98), but there was no statistically significant difference in burnout between nurses and physicians.

### Association Between Patient Safety Culture and WPV

After controlling for years of unit work experience, staff position, and type of work unit, patient safety culture was associated with all three forms of WPV (Table 3). Positive perceptions of teamwork across units and handoffs and transitions were associated with lower physical violence from a patient (odds ratio [OR] = 0.77, 95% CI = [0.63, 0.94]; OR = 0.78, 95% CI = [0.66, 0.93], respectively) and lower verbal abuse from a patient (OR = 0.72, 95% CI = [0.59, 0.88]; OR = 0.76, 95% CI = [0.64, 0.90], respectively). Positive perceptions of staffing and teamwork across units and a higher frequency of events reported were associated with lower verbal abuse from a visitor (OR = 0.84, 95% CI = [0.72, 0.99]; OR = 0.75, 95% CI = [0.62, 0.91]; OR = 0.88, 95% CI = [0.78, 0.99], respectively). However, positive perceptions of supervisor expectations and actions promoting patient safety were associated with higher physical violence from a patient (OR = 1.24, 95% CI = [1.03,1.48]). Positive perceptions of nonpunitive errors were associated with higher verbal abuse from a patient (OR = 1.22, 95% CI = [1.06, 1.40]) and a visitor (OR = 1.18, 95% CI = [1.03, 1.35]).

**Table 3.** Logistic Regression Analysis Examining the Association Between Patient Safety Culture and Workplace Violence Among Health Care Workers (*n* = 2,249)

Predictor variable	Physical violence from patient		Verbal violence from patient		Verbal violence from visitor	
	OR	95% CI	OR	95% CI	OR	95% CI
Years of work experience in the current unit	0.88	[0.80, 0.96]	0.91	[0.84, 0.99]	1.02	[0.94, 1.10]
Staff position						
Nurses	1.33	[1.02, 1.72]	1.76	[1.36, 2.27]	1.87	[1.44, 2.42]
Physicians	0.29	[0.18, 0.48]	1.22	[0.82, 1.82]	1.02	[0.67, 1.54]
Other	0.66	[0.44, 0.98]	0.75	[0.52, 1.08]	1.06	[0.73, 1.55]
Allied health (reference)						
Department						
Surgery	1.27	[0.90, 1.80]	0.68	[0.48, 0.95]	1.25	[0.90, 1.75]
Perioperative	0.76	[0.50, 1.15]	0.17	[0.12, 0.26]	0.40	[0.26, 0.62]
Obstetrics	0.05	[0.02, 0.14]	0.09	[0.06, 0.15]	0.38	[0.24, 0.60]
Pediatrics	2.43	[1.59, 3.72]	0.35	[0.23, 0.53]	1.59	[1.05, 2.41]
Emergency department	3.42	[2.22, 5.26]	4.80	[2.45, 9.41]	4.50	[2.88, 7.03]
Intensive care unit	1.57	[1.13, 2.20]	0.41	[0.29, 0.58]	1.34	[0.97, 1.85]
Psychiatric	2.06	[1.34, 3.18]	3.86	[2.12, 7.01]	1.01	[0.65, 1.55]
Others	0.73	[0.52, 1.02]	0.53	[0.39, 0.72]	0.92	[0.68, 1.26]
Medicine (reference)						
Patient safety culture^a^						
Supervisor/manager expectations and actions promoting patient safety	1.24	[1.03, 1.48]	1.06	[0.89, 1.27]	1.03	[0.87, 1.22]
Organizational learning–continuous improvement	1.04	[0.83, 1.30]	1.17	[0.94, 1.46]	1.10	[0.89, 1.37]
Teamwork within units	0.97	[0.81, 1.18]	0.86	[0.71, 1.04]	0.89	[0.74, 1.06]
Communication openness	0.92	[0.75, 1.12]	0.95	[0.78, 1.16]	1.05	[0.87, 1.28]
Feedback and communication about errors	1.02	[0.84, 1.25]	0.94	[0.77, 1.14]	0.93	[0.77, 1.12]
Nonpunitive response to errors	1.11	[0.97, 1.29]	1.22	[1.06, 1.40]	1.18	[1.03, 1.35]
Staffing	0.94	[0.80, 1.11]	0.99	[0.85, 1.16]	0.84	[0.72, 0.99]
Management support for patient safety	0.85	[0.72, 1.00]	0.89	[0.75, 1.05]	0.95	[0.81, 1.11]
Teamwork across units	0.77	[0.63, 0.94]	0.72	[0.59, 0.88]	0.75	[0.62, 0.91]
Handoffs and transitions	0.78	[0.66, 0.93]	0.76	[0.64, 0.90]	0.89	[0.76, 1.05]
Overall perceptions of patient safety	0.84	[0.69, 1.03]	0.87	[0.72, 1.06]	0.97	[0.81, 1.17]
Frequency of events reported	0.93	[0.82, 1.06]	0.96	[0.84, 1.09]	0.88	[0.78, 0.99]

*Note.* OR = odds ratio; CI = confidence interval.

a Agency for Healthcare Research and Quality Hospital Survey on Patient Safety Culture.

### Association Between Patient Safety Culture and Burnout

Multiple regression analysis indicated that the model was statistically significant (*p* < .001), accounting for approximately 43% of model variance in health care workers’ burnout (Table 4). Among the 12 dimensions of patient safety culture, seven dimensions contributed significantly and negatively to health care workers’ perceptions of burnout: organizational learning/continuous improvement (*B* = −0.09; *p* = .014); teamwork within units (*B* = −0.16; *p* < .001); staffing (*B* = −0.31; *p* < .001); management support for patient safety (*B* = −0.06; *p* = .023); teamwork across the unit (*B* = −0.07; *p* = .036); handoffs and transitions (*B* = −0.14; *p* < .001); and overall perceptions of patient safety (*B* = −0.09; *p* = .005). Overall, a better patient safety culture reduced health care workers’ burnout.

**Table 4.** Multiple Regression Analysis Examining the Association Between Patient Safety Culture and Health Care Worker Burnout (*n* = 2,211)

Predictor variable	*B*	*SE B*	β	*t*	*p*
Years of work experience in the current unit	0.01	0.01	0.01	0.69	.491
Staff position					
Nurses	0.09	0.04	0.05	2.11	.035
Physicians	−0.02	0.07	−0.01	−0.27	.784
Other	0.01	0.06	0.00	0.18	.860
Allied health professional (reference)					
Department					
Surgery	−0.03	0.06	−0.01	−0.46	.644
Perioperative	−0.05	0.07	−0.01	−0.71	.477
Obstetrics	−0.07	0.08	−0.02	−0.85	.397
Pediatrics	−0.01	0.08	0.00	−0.18	.856
Emergency department	−0.44	0.08	−0.11	−5.76	<.001
Intensive care unit	−0.09	0.06	−0.03	−1.56	.118
Psychiatric	−0.23	0.08	−0.05	−2.93	.003
Others	−0.15	0.06	−0.06	−2.64	.008
Medicine (reference)					
Workplace violence	0.21	0.04	0.10	5.79	<.001
Patient safety culture^a^					
Supervisor/manager expectations and actions promoting patient safety	−0.05	0.03	−0.04	−1.73	.084
Organizational learning/continuous improvement	−0.09	0.04	−0.07	−2.46	.014
Teamwork within units	−0.16	0.03	−0.12	−5.08	<.001
Communication openness	0.03	0.03	0.02	0.80	.426
Feedback and communication about error	−0.05	0.03	−0.04	−1.61	.107
Nonpunitive response to errors	−0.02	0.02	−0.02	−0.68	.495
Staffing	−0.31	0.03	−0.27	−11.39	<.001
Management support for patient safety	−0.06	0.03	−0.06	−2.28	.023
Teamwork across units	−0.07	0.03	−0.05	−2.10	.036
Handoffs and transitions	−0.14	0.03	−0.12	−4.82	<.001
Overall perceptions of patient safety	−0.09	0.03	−0.08	−2.84	.005
Frequency of events reported	0.00	0.02	0.00	0.02	.988

*Note.* Adjusted *R*^2^ = .44 (*N* = 2,211; *p* < .001). Job characteristics and workplace violence included as covariates. Workplace violence was defined as staff experienced (yes/no).

## Discussion

This study explored patient safety culture attributes that predict health workers’ experience of WPV and burnout. The study aimed to provide a foundation for promoting patient and worker safety and health by strengthening patient safety culture, consistent with current efforts to emphasize an integrated approach to patient and worker safety. Our results revealed that perception of patient safety culture, WPV, and burnout differed by occupational type and that patient safety culture significantly predicts WPV and worker burnout after controlling for years of work experience, staff position, and type of work unit.

### Patient Safety Culture

In our study, nurses and allied health professionals perceived patient safety culture more positively than physicians. This finding is consistent with prior studies in which nurses rated the culture of safety higher than physicians did (Blegen et al., 2010; Campbell et al., 2010), but inconsistent with other studies in which nurses had lower mean patient safety culture scores than both physicians and other health workers (Abrahamson et al., 2018; Famolaro et al., 2021). There are several possible explanations for differences in the perceptions of safety culture among health workers, including different job conditions, workloads, and safety training levels (Campbell et al., 2010; Willmott & Mould, 2018). A positive patient safety culture is critical because it increases the likelihood that health workers will engage in safe behaviors that protect patients, themselves, and others from risk. Future research is needed to investigate differing health care workers’ perceptions of patient safety culture to better target patient safety culture interventions and foster a shared responsibility in the promotion of patient safety.

### Patient Safety Culture and WPV

Higher levels of patient safety culture were associated with fewer experiences with WPV. Among the dimensions of patient safety culture, better teamwork across units predicted lower WPV incidents for all three forms of WPV, while better handoffs and transition predicted lower physical and verbal violence from patients. As reported in the literature, hospital stays can cause anxiety and fear in patients and their family members (Pellosmaa & Desouky, 2013). Occasionally, these feelings may place caregivers in unsafe situations if patients and family expectations are unmet due to poor coordination among health care workers (Najafi et al., 2018). For example, when interdepartmental or interprofessional collaboration is not well coordinated, miscommunication between health care workers and misunderstanding of roles and responsibilities can occur, which may result in care process delays or even adverse patient events (Najafi et al., 2018; Pompeii et al., 2020). These events constitute the primary reason health care workers are at risk of exposure to violence in the workplace.

Adequate staffing also predicted fewer health care worker reports of verbal violence from visitors. This finding confirmed previous research indicating that the risk of violence increases in understaffed situations, particularly during mealtimes and visiting hours (Occupational Safety and Health Administration, 2016). Although violence in the health care workplace is unlikely to be entirely eradicated, strategies and interventions for preventing potential violence are critical. Our findings indicate that situational factors that precipitate violence are similar to those that compromise patient safety and result in adverse patient experiences; thus, providing organizational supports and resources to enhance patient safety culture may be effective in ensuring the safety of both patients and workers.

Interestingly, higher levels of staff perceptions about supervisor/manager expectations and actions for promoting patient safety were associated with increased physical violence from patients, and nonpunitive responses to errors were associated with increased verbal violence from patients and visitors. These findings were unexpected and the lack of prior research made interpretation difficult. Additional research is needed to further explore these relationships.

### Patient Safety Culture and Burnout

A better patient safety culture was associated with lower worker burnout, which is consistent with prior research findings (Habibzadeh et al., 2020; Vogus et al., 2020). This finding indicates that improving patient safety culture can be an effective managerial strategy to reduce health care worker burnout. Adequate staffing was the strongest predictor of reduced burnout among the 12 dimensions of patient safety culture, followed by good teamwork within units and optimal handoffs and transitions. Active organizational learning for continuous improvement, good management support for patient safety, good teamwork across units, and a positive perception of overall patient safety were also significant predictors of lower levels of burnout.

As the health care field becomes more complex, specialized, and cooperative, the view of burnout has expanded from the immediate work context to organizational and management environment contexts, which include structural, social, cultural, and economic forces (Maslach et al., 2001). This study examined the risk factors for burnout in the cultural context of patient safety in health care organizations. Managerial values and efforts to improve patient safety may help workers to establish positive emotional and cognitive relationships with their work. Most previous studies aimed at reducing burnout have focused on individual-centered strategies, such as resilience training, stress management training, or mindfulness courses, rather than on organizational strategies to address burnout (Zhang et al., 2020), despite organizational factors playing a more significant role in burnout than individual factors in the workplace, where workers have less control over the stressors they encounter (Maslach et al., 2001). The most effective intervention approach is to combine organizational practice changes with individual strategies (Brooks Carthon et al., 2021; West et al., 2016). Further research is needed to design and test interventions that address critical organizational factors, including organizational culture, work environment, and administrative support, that contribute to burnout. Research addressing the identification and adoption of patient safety culture attributes that influence burnout will have a synergistic effect in improving patient safety and reducing worker burnout.

Our findings provide insights suggesting that hospitals may decrease worker burnout and prevent WPV by improving their patient safety culture at a systemic level. We found that teamwork across units and handoffs and transitions were common dimensions of patient safety culture that affected burnout and WPV. These domains emphasize hospital-wide cooperation across units. Health care delivery is inherently an interdependent and complex process that requires the collaboration of multiple teams (Rosen et al., 2018). Transitions of care (i.e., between hospital units or shift changes) require effective team interaction and communication skills to avoid the loss of critical information (The Joint Commission, 2017). A variety of studies have confirmed that teamwork and communication skills are necessary for quality health care delivery. When all health care staff operate cooperatively, health care teams can optimize patient outcomes, avoid medical errors, maximize productivity, and increase patient satisfaction (Rosen et al., 2018). Effective teams also create work environments that are more constructive, engaging, and resilient (Buljac-Samardzic et al., 2020). Because the well-being of health workers and the safety of patients develop interdependently in the context of teamwork (Welp & Manser, 2016), hospital administrators and managers should encourage all staff to participate in achieving patient safety goals and exercise strong leadership to facilitate collaboration between units.

Health care organizations are encouraged to assess their culture, provide leadership and feedback to employees, and implement interventions to reduce patient safety risks (Bienassis et al., 2020; The Joint Commission, 2012). Organizations are also urged to identify, mitigate, and address systemic issues that lead to physical, psychological, and emotional harm, including burnout, in workers and provide adequate resources to do so (NSC, 2020). Patient safety culture is at the heart of all of these efforts. Our findings on the connection between patient safety culture and the safety and health of workers can help health care institutions to develop better policies and practices aimed at promoting a healthy workplace.

There are a few limitations specific to this study. First, this study was conducted at a single, large academic medical center, which limits generalization; however, our data were gathered from all types of hospital workers who directly interact with patients rather than from specific professions. Thus, our findings may provide a more comprehensive understanding of how patient safety culture affects the safety and emotional health of health care workers. Second, because this study was cross-sectional in design, we cannot prove the causality of our findings. Additional research, including longitudinal or interventional designs, is needed to establish causal relationships.

## Implications for Occupational Health Practice

Workplace violence and burnout are significant factors that jeopardize the physical and psychological health and safety of health care workers. Despite widespread attention, these complex and problematic relationships remain difficult to resolve. This study’s findings suggest that occupational health nurses and practitioners can address these problems by highlighting the importance of improving patient safety culture to front-line caregivers, implementing relevant occupational health practices that address worker burnout, and, in turn, evaluating the impact of those changes on the safety and health of both patients and health care workers. The application of interventions that promote safety culture may have the greatest impact on employee health and safety, and on patient safety by creating an environment and organizational practices that encourage employee safety behaviors. Our findings provide hospital managers and occupational health professionals specific evidence to inform the development of strategies to protect workers from burnout and WPV. Furthermore, our results provide evidence to support the importance of an integrative approach to safety culture that fosters patient and worker safety and health.

**Applying Research to Occupational Health Practice**

Findings from this study indicate that high levels of patient safety culture are associated with low levels of WPV and health worker burnout. Thus, taking steps to promote key aspects of patient safety culture—greater teamwork within and across units, smoother worker handoffs and patient transitions, and adequate staffing—can motivate improvements to ameliorate WPV and health worker burnout. Also, applying a more integrated approach to patient–worker safety and health that recognizes the distinct connections between patient safety culture and worker safety and health can greatly improve working conditions. That is, the safety and health of workers and patients are inextricably linked; therefore, organizational leaders and occupational health professionals should adopt an integrated approach to worker and patient safety, health, and well-being that will guide future planning, implementation, and evaluation of innovations that drive improvements in the workplace.
